# Automation in canine science: enhancing human capabilities and overcoming adoption barriers

**DOI:** 10.3389/fvets.2024.1394620

**Published:** 2024-06-14

**Authors:** Nareed Farhat, Dirk van der Linden, Anna Zamansky, Tal Assif

**Affiliations:** ^1^Department of Information Systems, University of Haifa, Haifa, Israel; ^2^Department of Computer and Information Sciences, Northumbria University, Newcastle upon Tyne, United Kingdom; ^3^Lod Municipal Shelter, Lod, Israel

**Keywords:** automation, canine science, artificial intelligence, animal behavior, motion tracking

## Abstract

The emerging field of canine science has been slow in adopting automated approaches for data analysis. However, with the dramatic increase in the volume and complexity of the collected behavioral data, this is now beginning to change. This paper aims to systematize the field of automation in canine science. We provide an examination of current automation processes and pipelines by providing a literature review of state-of-the-art studies applying automation in this field. In addition, via an empirical study with researchers in animal behavior, we explore their perceptions and attitudes toward automated approaches for better understanding barriers for a wider adoption of automation. The insights derived from this research could facilitate more effective and widespread utilization of automation within canine science, addressing current challenges and enhancing the analysis of increasingly complex and voluminous behavioral data. This could potentially revolutionize the field, allowing for more objective and quantifiable assessments of dog behavior, which would ultimately contribute to our understanding of dog-human interactions and canine welfare.

## 1 Introduction

Dogs, *canis lupus familiaris*, are of increasing interest in many different disciplines, as can be witnessed by the increase in the scientific production on their cognitive and behavioral aspects (1). First of all, this interest can be attributed to the fact that dogs are useful clinical models for hundreds of human disorders. Indeed, they are large animal models, being physiologically and clinically more similar to human than other commonly used animal models, such as mice. Moreover, as companion animals also share the environmental conditions of their owners, similarly to humans they are affected by them. Numerous canine conditions are analogous to human diseases such as diabetes, cancers, epilepsies, eye diseases and autoimmune diseases, as well as rare diseases (2). Additional factors explaining dogs' popularity in science include fascination with the origins of dogs and their domestication, behavior, and cognition, as well as the need to better understand and regulate consequences of dog-human interactions and welfare, e.g., of working dogs and shelter dogs (1).

As a consequence of their living close to humans as pets, working or sheltered animals, dogs exhibit immense behavioral variability, stemming from their innate capacities as well as from environmental influence (3). Therefore, methods of *canine behavioral testing* are popular in research and practice. They are extensively used in cognitive science, veterinary science, working dog organizations, shelters for various purposes such as selection for breeding (4), learning abilities (5), prediction of suitability for work (6), adaptability in shelters (7, 8), animal models for human diseases (9), welfare (10).

Traditionally, data analysis in behavioral testing paradigms is done through direct and systematic human observation (11, 12) in a process where behaviors are defined in precise terms (usually they can have types of either event or state), deciding on the type of measurement, sampling method, etc. Properly trained human observers can typically provide accurate measures of almost any behavior. However, relying on human observation imposes severe limitations on behavioral data acquisition and analysis. As highlighted by Anderson and Perona (13), it is firstly a laborious and tedious task, limiting the volumes of processed data, as well as the number of analyzed behaviors or behavioral variables. But even more importantly, human analysis of behavior is prone to *subjectivity*, strongly depending on human perceptual abilities, leaving room for human error. Moreover, human understanding and interpretation of behavior is in itself subjective and sometimes inconsistent.

Advances in artificial intelligence (AI) open the door to new exciting opportunities to overcoming these limitations. Automated methods in human applied behavior research are already revolutionizing the field (14), as they can provide increased precision of measurement across smaller temporal units and for longer periods of time compared with human observers. These advancements have significant implications for understanding human behavior (15), mental health (16), and cognitive processes (17).

In the animal domain the need for promoting more objective and quantifiable assessment and measurement of behavior is also well-acknowledged [cf. (18–20)], pushing what is referred to “computational animal behavior analysis” (CABA) (13, 21, 22), also referred to as “behavioral imaging” (23). The release of deep learning frameworks such as DeepLabCut (24) has unleashed the potential of video-based motion tracking and pose recognition in many animal species. Additional tools such as EZtrack (25), LEAP (26), DeepPoseKit (27), idtracker.ai (28) provide more light-weight options, and support advanced settings such as large group tracking. An additional step in the field of CABA is taken by the paradigm shift from two-dimensional to three-dimensional data using multi-view cameras, enhancing our abilities to track every single point on the animal's body and addressing full behavioral repertoires of various species. Large scale projects employ systems integrating multiple camera views to allow continuous 3D tracking of freely behaving animals, such as CAPTURE (29) and CaT-z (30) for rodents, and Open Monkey Studio (31) for rhesus macaques.

In canine science, however, the adoption of automation for data analysis has been quite slow. One reason for this is that the generic deep learning platforms discussed above are not easily adaptable from controlled laboratory environments. For instance the JAABA system (32) which allows the user to annotate a small set of data to train a model specific to the study and is likely to perform poorly on dogs due to the diversity of morphologies and breeds. DeepLabCut (24) has been recently utilized for canine pose detection for emotion recognition (33) and recognition of stress related behaviors (34); both these studies noted the limitations of the system due to breed diversity.

The lack of tools tailored for the canine domain leads to the need for self-developed, domain-specific systems, which in its turn implies the necessity for multidisciplinary collaborations, leading to differences in terminology, research methods and expectations of different stakeholders. We explore in this study, this and other human-related adoption barriers for automation in the field.

The growing interest in automation in canine research, makes this a timely moment for a reflection and systematization of processes in this domain. What is usually presented in scientific papers applying automation in dog behavior analysis are just the end results, with the process of getting to them being left out of scope. Scientific papers focusing on the use of automation in analyzing dog behavior often only report the final outcomes, typically omitting the detailed process that led to these results. However, these details are essential to evaluate the insights gained and to explore future directions in this field.

This paper aims to promote automation in canine science by gaining a better understanding not only into its current usage, but also into *barriers* toward a wider adoption, and by scrutinizing not only artifacts of such analysis (i.e., study results), but also the *ways* to obtain them. To this end, we address the following research questions:

- How is automation currently used in canine science for dog behavior analysis?
- What are the challenges and barriers toward a wider adoption of automation in this context?

To answer the first question, we provide a comprehensive review of *N* = 16 studies that have applied some kind of automated analysis in the context of dog behavior. We dissect and categorize the reviewed works, identifying important dimensions, which represent the way automation is used today in this field. To address the second question, we perform an empirical study with *N* = 24 researchers who have experience with applying automation in the field, scrutinizing their perceptions and attitudes of the integration of automation and potential barriers toward their wider adoption.

The remainder of this paper is structured as follows: Section 2 encompasses a comprehensive review of studies that implement automated analysis in the realm of canine behavior. In Section 3, we present an empirical inquiry into the perceptions and attitudes of researchers in an effort to clarify the challenges impeding the broader adoption of automation in this field. The paper concludes with a discussion.

## 2 A mapping of automated approaches in dog behavioral data analysis

To promote systematization, as well as to provide an overview of the automated analysis methods that are relevant for animal behavior analysis outside of laboratory settings, we survey in this section studies that apply automated analysis in the form of machine learning techniques of some type.

### 2.1 Review protocol

#### 2.1.1 Search strategy

We conducted the literature search using a snowballing approach (35) employing Google Scholar, as it has a broad reach covering most academic databases to avoid publisher bias, *and* because it includes relevant gray literature and pre-prints, which is particularly relevant to include in literature reviews of novel fields where new approaches are rapidly developed and published online.

We used the following query in Scholar to identify a first set of relevant core papers:

(animal OR dog OR canine AND (automated OR machine learning OR deep learning OR artificial intelligence OR ML OR AI) AND (behavior recognition OR behavior analysis)

The chosen keywords were selected to ensure a comprehensive and inclusive search. The terms “animal”, “dog”, and “canine” were used to focus on studies specifically related to dogs. The terms “automated”, “machine learning”, “deep learning”, “artificial intelligence”, “ML”, and “AI” were picked to capture all studies utilizing these computational methods in their research. Finally, “behavior recognition” and “behavior analysis” were included to focus on studies that apply these automated methods to behavior analysis or recognition, which is the core subject of our review.

We then identified a number of core papers, which we then conducted forward and backwards snowballing from, reading through both the literature cited and citing these works. We continued this approach by identifying relevant works using the selection criteria below, and for each identified relevant work, again snowballing by reviewing the cited and citing literature to identify further relevant literature to include.

We stopped the snowballing when we no longer identified any potentially relevant works to assess in the lists of works citing or cited by the selected papers.

#### 2.1.2 Selection criteria

We liberally applied the following inclusion criteria while reading papers:

- **inc1** The paper applies an automated approach in dog behavior analysis;

and then refined the selection with these more concrete exclusion criteria:

- **exc1** The paper does not apply the automated approach in the context of a concrete question related to dog behavior, health, welfare, cognition, etc. Thus, papers which solely design or propose new computational methods were excluded.
- **exc2** The approach is not applied on animal data (e.g., human behavioral data, or animal data obtained by human scoring).

#### 2.1.3 Data extraction and dissection

We extracted the following data from the included papers (see Supplementary Table 1):

- What is the general domain/topic of the study, in the context of which the behavior is analyzed? The topics include dog-human relationships, health, welfare, etc.
- What are the concrete research questions explored in the study?
- How was the data obtained?
- How is behavior quantified, measured or computationally represented?
- What are the extracted features from the behavior representation?
- How are the extracted features used to answer the research questions?

The first author extracted the data from the included papers, which another author independently verified.

### 2.2 Findings

#### 2.2.1 Selected papers

Supplementary Table 1 presents a table of the 16 selected papers. Four of them address the topic of dog-human relationships (36–39), five of them focus on clinical behavioral aspects, of which three focus on ADHD-like behavior (40–42), one on separation anxiety (43), and one on ataxic gait patterns (44). Three of them focus on automation of scoring and assisting in behavioral testing (45–47), an additional three on emotion recognition (33, 48, 49), and one on shelter dog welfare (50).

#### 2.2.2 Quantification of behavior

**Figure 1:**
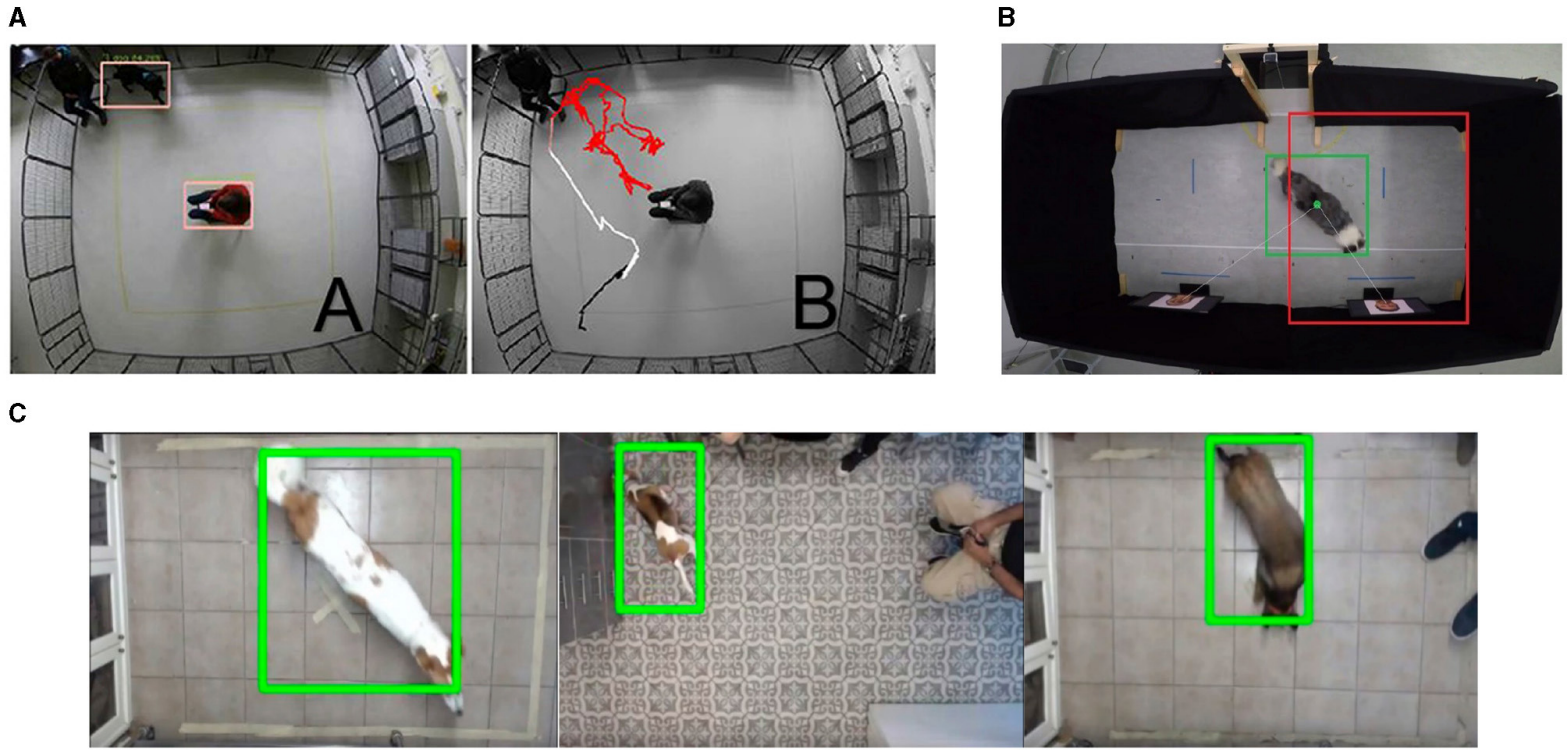
Examples demonstrating the application of a convolutional neural network to generate a 2D time series representation of the dog from the selected papers. **(A)** Menaker et al. (45): detection of dog and person and the resulting trajectory. **(B)** Karl et al. (36): detection of dog. **(C)** Bleuer-Elsner et al. (41): detection of dog. Reproduced with permission.

The idea behind automation of behavior analysis in these studies is in transforming obtained raw data into some computational representations of behavior that can then be manipulated either by machine learning or statistical methods. In most collected works, the data is obtained as visual data (videos or images) and needs to be manipulated. The most common representation of behavior used in the majority of these works is by tracking the dog's body (either in two or three dimensions). Bleuer-Elsner et al. (41), Karl et al. (36), Byosiere et al. (50), Fux et al. (40), Menaker et al. (51), Farhat et al. (46), Tsiourti et al. (47), and Watanangura et al. (42) use a convolutional neural network for object detection, producing a time series representing a trajectory of the dog from an above view, see examples in Figure 1. Similarly, Völter et al. (37, 38) and Ren et al. (39) use a convolutional neural network on multiple cameras producing a 3D time series representation of multiple key points on the dogs' bodies. More subtle behavioral representations include Ferres et al. (33) which uses a convolutional neural network for landmark detection, producing up to 24 points on the dogs' body in images, and Boneh-Shitrit et al. (48) which uses deep learning to extract both facial action units and deep learning learnt features from frontal images of dog faces. The remainder of works obtain acceleration and angular velocity measurements of movement from the accelerometer and gyroscope sensors on the dog's body. Aich et al. (49), Wang et al. (43), and Engelsman et al. (44) format the collected signals as 3D time series of the dog's body parts.

#### 2.2.3 Extracted features

As highlighted in (52), a major focus in computer vision is on the use of *features* that can be extracted from images. Features can be hand-crafted, or manually designed, or learnt, as is the case in deep learning paradigms. Most of the studies reviewed here represent behavior in the form of time series or trajectories. Some of them extract from these trajectories some high-level meaningful features, such as average speed, residence in areas of interest, distance from and interaction with certain objects or people, studies that used time series of key points additionally extracted specific limb movement such as head angle, tail angle, velocity and amplitude. Two studies (46, 48) used deep learning to extract features automatically from the computational representation of the behavior.

#### 2.2.4 Answering the research question: statistical testing vs. machine learning

The way in which (automatically) extracted features are used for addressing a biologically meaningful research question deserves special attention, as this point is not sufficiently studied in animal behavior research. Traditionally in this research a hypothesis is formed, for which animal behavior is measured (either by manual coding, or in the more novel automated approaches discussed here). The measurements are then used for statistical testing of the hypothesis. However, the integration of the machine in data analysis means that we can use a different strategy. Machine learning classification is an alternative, powerful approach which is not discussed enough in the scientific community addressing animal behavior.

Li and Tong (53) discuss the differences between the two approaches of statistical testing vs. machine learning classification, which are rooted in two different cultures of inference vs. prediction. Inferential tasks aim to infer an unknown truth from observed data, and hypothesis testing is a specific framework for doing so. Prediction tasks, on the other hand, aim to predict an unobserved property of an instance based on the available (observed) features of that instance. Such prediction relies on building a prediction rule from the features to the unobservable property of interest, either based on human knowledge, or established from data.

#### 2.2.5 The automation process: from quantification of behavior to answering research questions in behavior

**Figure 2:**
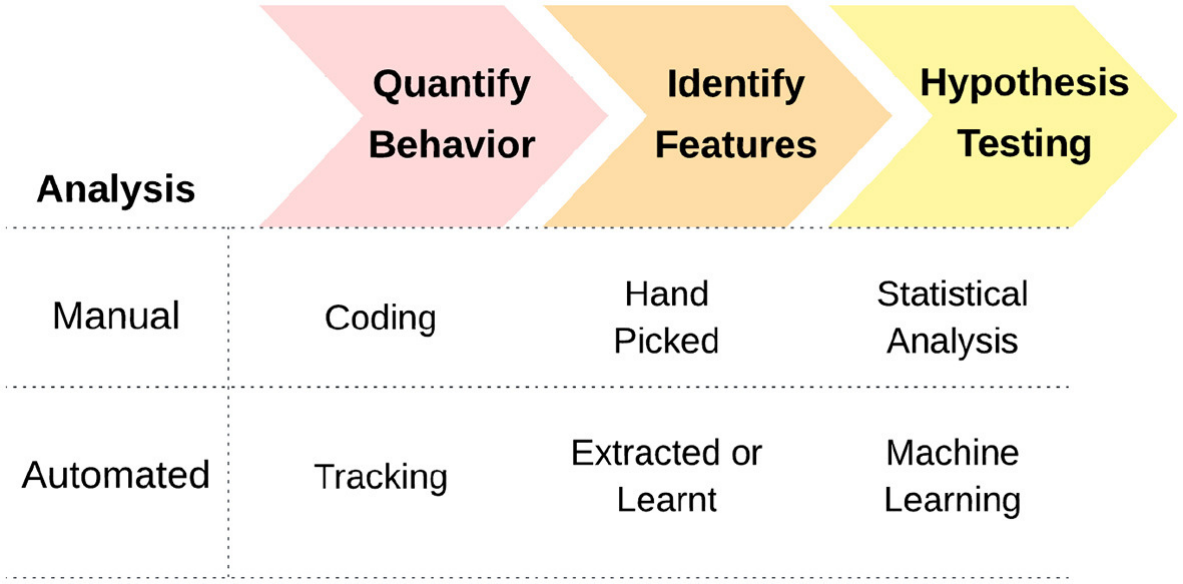
The proposed conceptual model of the process of automating behavior analysis.

We now present a conceptual model of the process of automation emerging from our analysis of the studies reviewed here. The steps involved in automated analysis in these works are presented in Figure 2. They are typically the following: (1) quantification of behavior, (2) extraction of features, and (3) using these features to answer some biological question related to behavior. Each of these steps can be done manually, or involving automation. Traditional ethological analysis keeps all of these stages manual: coding behavioral categories, quantification and then statistical analysis.

**Figure 3:**
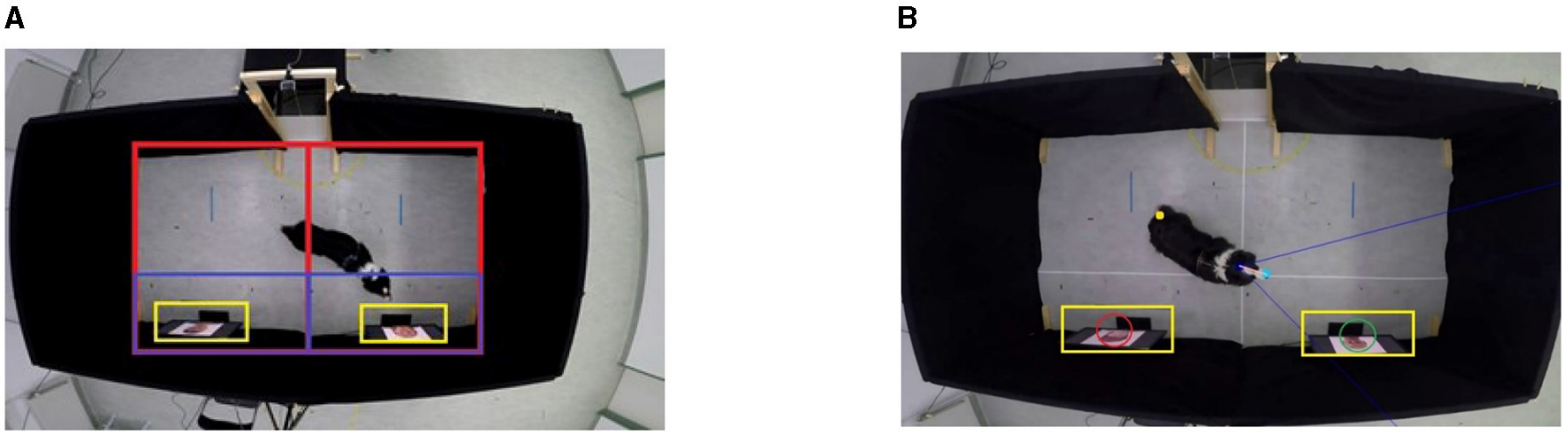
Examples of hand-picked features in Karl et al. (36). **(A)** Defined areas of interest that were used to calculate time of residence. **(B)** Example of participant field of view calculation. Reproduced with permission.

Below, we explore how the reviewed works make use of automation at each of these steps:

1. Bleuer-Elsner et al. (41) and Fux et al. (40) addressed the analysis of ADHD-like behavior in dogs. Dogs were recorded moving freely during a behavioral consultation visit in a veterinary clinic, with some diagnosed with ADHD-like behavior and others in a control group with no reported behavioral problems. *RQ:* Are there differences in behavior in the consultation room between dogs diagnosed with ADHD-like behavior and control group dogs? *Behavior Quantification:* 2D time series (trajectory) of the dog was extracted. *Feature Extraction:* The features extracted were hand-picked (traveled distance, average speed, straightness, intensity of use, etc.)*Answering the RQ:* A machine learning model was developed to separate between the two types of dogs' behaviors.
2. Aich et al. (49) explored the feasibility of using wearable sensors for analyzing activity and emotional patterns of dogs. Data was collected using sensors placed on the necks and tails of the participants *N* = 10, while preforming seven distinct activities and three emotional states (positive, neutral and negative). These sensors, equipped with accelerometers and gyroscopes, measured linear and rotational motions in all directions. *RQ:* Can machine learning techniques recognize activity and emotional patterns of dogs from wearable sensors?*Behavior Quantification:* 3D time series of the head and tail, received from the wearable sensors. *Feature Extraction:* The extracted features were hand-picked (statistical and peak based features). *Answering the RQ:* Two machine learning models were developed, the first detects the activity, the second detects the emotional state.
3. Karl et al. (36) investigated the engagement of an attachment-like system in dogs when they see human faces. Stimuli in the form of videos of the caregiver, a familiar person, and a stranger were presented to the dog participants *N* = 24, showing either happy or angry facial expressions. *RQ:* What are the neural, visual, and behavioral responses of dogs to human faces, and how do they differ between caregivers, strangers, and familiar persons?*Behavior Quantification:* 2D time series (trajectory) of the dog was extracted. *Feature Extraction:* The extracted features were hand-picked (time of residence in areas of interest, distance from screens, field of view, see Figure 3).*Answering the RQ:* Statistical analysis was used to test the differences between conditions.
4. Byosiere et al. (50) examined the behavior of shelter dogs before COVID-19 pandemic restrictions and during the restrictions for a period of two weeks each. The participants *N* = 34 were recorded in their kennels for 15 seconds each hour in daytime. *RQ:* Are there differences in shelter dogs activity levels before and during the COVID-19 pandemic?*Behavior quantification:* 2D time series (trajectory) of the dog was extracted. *Feature extraction:* The extract feature was hand-picked (step count; which is a quantification of the participants' movement). *Answering the RQ:* Statistical analysis was used to test the differences between conditions and times of day.
5. Ferres et al. (33) addressed the different dog emotional states and detecting them based on posture analysis of images. Four emotional states were used (anger, fear, happiness, and relaxation) *RQ:* Can machine learning techniques recognize emotional state of dogs from body language in images? *Behavior quantification:* 24 key points on the dogs' body. *Feature extraction:* The extracted features were hand-picked pose metrics (tail position, weight distribution, mouth condition etc.). *Answering the RQ:* Machine learning models (neural network on landmarks, and a classification tree on the extracted features) were used to classify the different emotional states.
6. Völter et al. (37) aimed to investigate whether dogs are sensitive to the intentions underlying human actions using the unwilling-unable paradigm. The study involved two pre-registered experiments, the first, a within-subject design where the participants *N* = 48 observed a human actor who either intentionally (teasing) or unintentionally (clumsiness or blocked area) failed to provide a treat. In the second experiment, participants *N* = 48 observed two different human actors who performed either a clumsy or teasing demonstration of attempting to provide a treat. *RQ:* Are dogs sensitive to the intentions underlying human actions? *Behavior quantification:* 3D time series of four key points on the dog's body (snout, head center, base of tail, and tip of tail). *Feature extraction:* The extracted features were hand-picked (visited areas, tail angel, visiting caregiver). *Answering the RQ:* Statistical analysis was used to test the differences between the dog's reaction to the experimenters' intentions.
7. Menaker et al. (45) explored the automatic analysis of dogs' behavior in a behavioral test. Participating dogs *N* = 30 were recorded during a stranger test, which allowed the dog to move freely in a room with a stranger. *RQ:* What is the potential use of unsupervised learning for pattern discovery dog behavior during a stranger test?*Behavior quantification:* 2D time series (trajectory) of the dog and stranger were extracted. *Feature extraction:* The chosen features were hand-picked (speed, covered movement area, approaching test person etc.). *Answering the RQ:* Machine learning (k-means clustering) was used to identify dogs with similar behavior in the stranger test.
8. Boneh-Shitrit et al. (48) explored the recognition of dog emotional state, comparing the results of facial action units and deep learning techniques. Participating dogs *N* = 29 were recorded in a controlled experiment, inducing negative (frustration) and positive (anticipation) emotional states using treats. *RQ:* Can machine learning techniques recognize emotional state of dogs from facial expression?*Behavior quantification:* Facial action units *Feature extraction:* The extracted features were automatically learned from the video using deep learning. *Answering the RQ:* Machine learning models (neural network on extracted features, tree based models on facial action units) were used to classify the different emotional states.
9. Ren et al. (39) investigated the effects of social cues on tail wagging during dog-human interactions. The participating dogs *N* = 10 were recorded over three consecutive days, interacting with the experimenter for 5 minutes (neutral postured experimenter, provided treats without direct contact). *RQ:* How does the dog's tail wagging behavior during dog-human interactions manifest, and what are the underlying neural and behavioral mechanisms of this behavior? *Behavior quantification:* 3D time series of four key points on the dogs' body (withers, back, croup, and tail tip). *Feature extraction:* The extracted features were hand-picked (tail angel, amplitude, and velocity).*Answering the RQ:* Statistical analysis was used to test the differences in tail wagging behavior across the test days.
10. Wang et al. (43) aimed to monitor and detect dog separation anxiety symptoms using wearable sensors. The sensors, placed on back and neck, were used to monitor home-alone cage-free *N* = 8 participants to identity behavior patterns that indelicate separation anxiety symptoms. *RQ:* Can machine learning detect and manage separation anxiety in dogs? *Behavior Quantification:* 3D time series of the head and body, received from the wearable sensors.*Feature Extraction:* The features were automatically extracted using machine learning models (head posture and body posture events).*Answering the RQ:* Machine learning (Complex event processing and fuzzy logic) was used to classify the dogs' behavior pattern as normal or abnormal, the latter indicating a symptom of separation anxiety.
11. Engelsman et al. (44) investigated the use of smartphone sensors to measure ataxic gait patterns in dogs. The sensor was attached to the participating dog's back using a harness, and the dog was then walked on a leash at a steady pace 5 times. Which resulted to the capture of 770 walking sessions of *N* = 55 healthy dogs, and *N* = 23 dogs with ataxia. *RQ:* What is the feasibility of using body-worn smartphone sensors to automatically classify between dogs diagnosed with ataxia and a healthy control group? *Behavior quantification:* 3D time series of the dog, using body worn sensor.*Feature extraction:* The extracted features were hand-picked statistical and frequency features.*Answering the RQ:* Machine learning was used to classify the gait patterns as either healthy or ataxic.
12. Völter et al. (38) examined the behavioral change of dogs N=37 exploring a room with new objects in the presence of the owner and/or stranger. *RQ:* How does separation from their caregiver, and presence of a stranger, affect dogs' exploratory behavior in a novel environment?*Behavior quantification:* 3D time series of eight key points on the dog's body (snout, head center, right ear, left ear, base of neck, hip region, tail base and tail tip). *Feature extraction:* The extracted features were hand-picked (residence in areas of interest, traveled distance, distance from objects, field of view, and tail angel). *Answering the RQ:* Statistical analysis was used to test the differences between the dog's behavior in presence and absence of owner.
13. Farhat et al. (46) investigated a computational approach to assess behavioral traits. The participated dogs N=53 were recorded in a behavioral test reacting to the presence of a strange, their coping styles were categorized into neutral, negative (reacting away from the stressor), and positive (reacting toward the stressor) reactions. *RQ:* Can the machine learning techniques identify different behavioral profiles in an objective, human-free way in the stranger test? *Behavior quantification:* 2D time series (trajectory) of the dog and stranger was extracted. *Feature extraction:* The chosen features were automatically learned using machine learning (unsupervised clustering). *Answering the RQ:* Machine learning models were used to classify dogs to the different behavior groups.
14. Tsiourti et al. (47) *RQ:* Do dogs use logical reasoning or associative learning to solve an invisible displacement task?*Behavior quantification:* 2D time series (trajectory) of the dog was extracted. *Feature extraction:* The chosen features were hand-picked (pace, travel straightness, covered area). *Answering the RQ:* Statistical analysis was used to test the differences between the dogs' travel based on placement type (visible/invisible) and object (toy/food).
15. Watanangura et al. (42) investigated the effects of fecal microbiota transplantation (FMT) on behavioral comorbidities in a dog model of epilepsy. Participants *N* = 9 underwent FMTs from a donor with controlled epilepsy three times, two weeks apart. Follow-ups were conducted at three and six months post-FMTs. Various evaluations were performed, including behavioral analysis and a range of biological tests. *RQ:* Can FMT improve behavioral comorbidities and cognitive dysfunction in dogs with drug-resistant epilepsy? *Behavior quantification:* 2D time series (trajectory) of the dog (and owner and/or stranger when relevant) was extracted. *Feature extraction:* The extracted features were hand-picked (time spent in areas of interest, movement area, speed etc.). *Answering the RQ:* Statistical analysis was used to test the FMTs effect overtime in the various follow-up tests (behavioral and biological).

### 2.3 Conclusions

The aim of this review was to assess the existing landscape of automation applications in canine science for dog behavior analysis. We have mapped the current situation with respect to how automation is currently utilized, analyzing 16 studies applying state-of-the-art automation in the context of dog behavior analysis. A conceptual model consisting of three steps arose from our analysis: (i) quantification of behavior, (ii) feature extraction, and (iii) hypothesis testing/answering the research question (either using statistical analysis or machine learning).

Overall, we observe a tendency to use straightforward and basic techniques at both stages (i) and (ii). In particular, a significant portion of the studies we reviewed utilized relatively simple object tracking methods for quantifying behavior [stage (i)]. However, there is a vast array of more sophisticated techniques in computer vision–like landmark localization (54), activity recognition (55), segmentation (56), and zero-shot learning (57) that hold great potential for this field. These more advanced approaches are yet to be fully explored and harnessed in the future.

Furthermore, most of the studies we reviewed tend to select a restricted range of features by hand (stage (ii)). Although these features are chosen based on expert knowledge in the field and can be quite informative, this method has its limitations. Specifically, the narrow scope of these hand-picked features might not provide the comprehensive data necessary to fully address the research questions at hand. Using alternative approaches in machine learning, such as deep learning, automated feature selection and unsupervised learning may be beneficial in this domain [see, e.g., the approaches used in (46, 48)] and should be further explored.

It is also notable that testing the hypothesis [stage (iii)] is most commonly addressed by statistical methods, following traditional approaches in behavior research (58). However, some recent studies have applied machine learning techniques.

### 2.4 Threats to validity

In conducting this literature review, several potential threats to validity were considered, acknowledging the need for a critical assessment of the findings.

- *Incomplete retrieval of studies*: despite thorough search strategies, there is a risk of incomplete retrieval. Cross-referencing citations and consulting multiple databases were employed to minimize this threat.
- *Inappropriate or incomplete search terms in automatic search*: the procedure of determining the search query was based on iterative refinement using identified papers as example inputs.
- *Review process:* this review is not a systematic review, and as such, the inclusion of studies may not follow a strict protocol. While efforts were made to conduct a comprehensive and thorough review, the absence of a formal systematic approach introduces a limitation in terms of standardized study selection.

## 3 Barriers to adoption of automation: an empirical study

To investigate the perceptions of animal researchers toward automated analysis tools, an exploratory study (59) was designed. This group consisted of researchers working on both fundamental and applied animal behavior research, excluding those in laboratory settings, and having at least some experience with dog behavior. The study adopted a mixed-method approach, incorporating both qualitative and quantitative approaches, as detailed in the “Procedure” subsection. The experimental design was reviewed and approved by the Ethical Committee of the University of Haifa. Informed consent was obtained from all participants. All relevant institutional guidelines and codes of conduct for ethical research were followed.

### 3.1 Participants

Twenty-four animal researchers were recruited via an invitation to participate in our study posted on an active international Facebook group on animal-centered computing, and by using the authors' own networks. To ensure privacy, we did not collect any personal information that could be used to identify any demographic subgroups. All participants volunteered to take part in the study and did not receive any compensation. Table 1 provides an overview of the participants' backgrounds, the species they currently investigate, and their years of experience in their respective fields.

**Table 1:** Overview of the backgrounds of the animal researchers, the species they investigate, and their years of experience in their respective fields.

**ID**	**Background**	**Species currently investigating**	**Years of experience**
P1	Cognitive science.	Laboratory animals.	5–10 years
P2	Animal behavior.	Companion animals.	10+
P3	Veterinary science.	Companion animals.	10+
P4	Veterinary science.	Companion animals.	0–2 years
P5	Veterinary science.	Companion animals.	10+
P6	Animal computer interaction.	Various.	5–10 years
P7	Animal behavior.	Companion animals.	10+
P8	Animal behavior.	Companion animals.	10+
P9	Computer science, neuroscience.	Dogs, horses, zoo animals.	10+
P10	Animal behavior.	Companion animals.	5–10 years
P11	Computing and design.	Various.	5–10 years
P12	Founder, dog trainer.	Companion animals.	5–10 years
P13	Veterinary science.	Companion animals.	0–2 years
P14	Computer science, robotics.	Companion animals.	3–5 years
P15	Veterinary science.	Companion animals.	5-10 years
P16	Neuroscience.	Laboratory animals.	3–5 years
P17	Cognitive science.	Companion animals.	0–2 years
P18	Animal behavior.	Companion animals.	10+
P19	Animal behavior.	Companion animals.	5–10 years
P20	Veterinary science.	Companion animals.	0–2 years
P21	Veterinary science.	Companion animals.	3–5 years
P22	Animal behavior.	Dairy calves.	0–2 years
P23	Animal behavior.	Companion animals.	10+
P24	Animal behavior.	Chickens.	3–5 years

### 3.2 Procedure

The data collection instrument was designed as a questionnaire incorporating both Likert-like scale and open-ended questions, designed to gather comprehensive insights into participants' experiences and perceptions. The formulation of questions drew from the authors prior involvement in collaborations with animal researcher (36, 46, 50). Furthermore, the questionnaire underwent a pilot phase, involving a behavioral veterinarian who had experience with an automated tool, and an animal behavior researcher who had not previously utilized an automated analysis tool. The valuable feedback provided by both participants contributed to the refinement and improvement of the questionnaire's content.

We structured the questionnaire into multiple sections to examine both current experience and future perspective on automation in the context of animal research. Concretely, we elicited the following data:

- *Section 1: Background information*. Inquires about participants' background, the animal species they study, their years of experience in animal behavior, and their previous experience using automated tools for data analysis.
- *Section 2: Experience with automated tools*. Asks participants about their experience with automated tools for data analysis, the reasons for choosing automated tools, their satisfaction with the results achieved, challenges faced while collaborating with data scientists, and how the outcome compared to their initial expectations.
- *Section 3: Issues and challenges*. Explores participants' confidence in understanding the precision of the automated analysis tool, their trust in the outcomes produced by the tool, steps data scientists can take to help participants understand and trust the results, difficulties in communication with data scientists, preprocessing required for data preparation, benefits of automated analysis for research, and preference for non-automated analysis methods.
- *Section 4: Reflection on the future of automation in animal behavior research*.: Asks participants about barriers to wider adoption of automated analysis tools, suggestions to overcome these barriers, likelihood of using automated tools in future research, and use of other technologies to assist in previous projects.

The full questionnaire can be found in Supplementary Data Sheet 1.

### 3.3 Data analysis

We obtained 24 responses to the questionnaire, which were analyzed using thematic analysis (60). All authors individually familiarized themselves with the data. Following on from this, multiple collaborative sessions were held where all authors discussed and framed interpretations of the data to propose and refine key themes focused on the challenges of adopting automation in animal behavioral data analysis, with a particular focus on dogs.

The first author coded the qualitative data obtained in the questionnaire in two iterations, identifying repeated meaningful patterns and using this to construct an initial classification framework for the data. This framework was then applied to the data in a similar iterative fashion and discussed with all authors. In total, we constructed three core themes that encapsulated the different codes.

### 3.4 Findings

#### 3.4.1 Descriptive analysis

**Figure 4:**
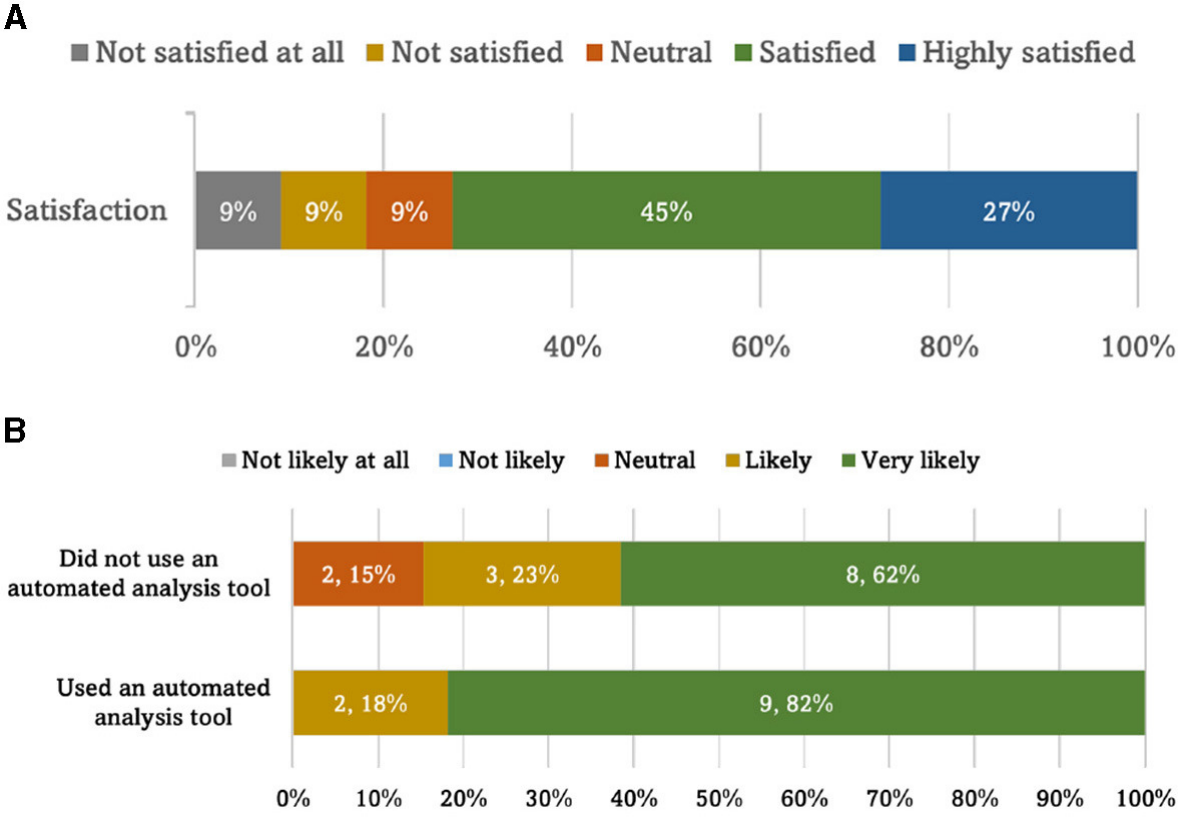
**(A)** Participants' satisfaction with the achieved results from an automated analysis tool. **(B)** The likelihood of participants using an automation data analysis tool in future work divided by their previous use of an automated analysis tool.

When queried about their prior experience with automated data analysis tools, 11 respondents (46%) affirmed previous use, while the remaining 13 (54%) indicated otherwise. Figure 4A provides an overview of the satisfaction levels reported by participants who have employed these automation tools, reflecting on their achieved results. Figure 4B presents the participants' anticipated likelihood of incorporating automated data analysis tools into their future work.

#### 3.4.2 Thematic analysis

The thematic analysis of the qualitative data yielded two prominent themes, which we explore next.

**Automation is useful for overcoming human limitations**

One important motivation for opting to work with automated tools for analysis of behavioral data, mentioned by some participants, was **the need to increase accuracy and objectivity of analysis** [“*My interest was to find an objective measurement tool”* (P5); “*I was looking for objectivity”* (P8); “*It is the most accurate way I have heard of for tracking multiple animals.”* (P24)].

Another reason was **the necessity to work with large volumes of data and reducing time for manual labor** [“*I needed to do a huge amount of tracking behavior in limited time”* (P7).; “*It removed hundreds of hours of work and was reapplied to nearly a dozen experiments.”* (P16)].

Perhaps the most interesting reason for using automated analysis as revealed by several participants, was **the need for an analysis that could not (or are extremely hard to) be performed manually** [“*Without the use of automated analysis, we would not have been able to calculate the main behavioral parameters of interest for our experiment.”* (P14)]. Specific examples of such parameters are:

- Speed of movement [“*We wanted to analyze behavioral measurements (specifically speed of movement of a dog) that were very hard to calculate ‘manually' though video observation. Therefore we resorted to the use of an automated video analysis software.”* (P14)].
- Length of trajectory [“*We wanted to code travel length of free-moving dogs in an open field, which would have been very difficult manually.”* (P10)].
- Approximating a dog's visual field [“*We needed a sophisticated analysis, e.g. dogs visual field.”* (P23)].

**However, human limitations also challenge the adoption of automation**

The majority of the barriers challenging the wider adoption of automation were discovered to be human-related. Among the barriers that have been mentioned by participants for a wider adoption of automation in the field of animal behavior analysis, particularly dominant were **lack of awareness to existing tools** and **a steep learning curve**. The **complexity of computational tools** is another issue, which is closely related to the latter [“*... tools should be simple and minimal and not try to cover every variation. They can become a lot more complicated to learn and there are more issues that could come up which we may not realize when building it.”* (P16)].

Suggestions to overcome these barriers included enhancing education on computational topics; some interesting suggestions were:

- “*Courses organized for universities/post grad schools/animal behavior departments”* (P15),
- “*Animal Behavior courses should teach programming in a fun way! Make it less daunting to learn. Also easier no-code tools are now possible, esp with LLMs”* (P17).
- “*Make the technology more accessible and more outreach/demos on how they work.”* (P21).

An important factor mentioned by our participants was also **communication gaps** between people from different disciplines, which can be overcome by having more discussions about the tools and the implicit assumptions made about them:

- “*There is often a communication gap between the authors of the tool and researchers, making the tools harder to adopt and leading to potential problems where the researchers don't know what went wrong and may think the entire tool doesn't work or they aren't qualified.”* (P10).
- “*Being aware about different hidden assumptions of the collaborators and talking about those explicitly is helpful.”* (P14).

The **availability of adequate funding** is another obvious issue contributing to the limited adoption of automation in the field of animal behavior. [“*... budget not obtained for this research.”* (P1), “Price of software capable of this ...” (P21), “*... cost or lack of university subscription.”* (P22)].

### 3.5 Threats to validity

This section addresses potential threats to the validity of the exploratory study. The examination of these threats aims to provide a transparent acknowledgment of limitations associated with the design, execution, and findings of this section.

- *Questionnaire Formulation:* The formulation of questionnaire questions by the same author introduces a potential bias in the inquiry process. Despite piloting for understandability, the selective framing of questions may have overlooked relevant obstacles, potentially affecting the comprehensiveness of participant responses.
- *Selective Inquiry:* Deliberate crafting of questions focusing on challenges in utilizing automated analysis tools might inadvertently neglect other pertinent obstacles. This selective inquiry may introduce response bias, limiting the our ability to capture a comprehensive view of participants' experiences.
- *Participant Pool and Recruitment:* The generalization of results is constrained by the relatively small number of participants, and the recruitment from social circles and collaborative networks. A larger and more diverse sample is essential for enhancing the validity of the findings and ensuring broader generalizability.

## 4 Discussion

The aim of this work was to advance the application of automated techniques in the field of canine science. We have mapped the current situation with respect to how automation is currently utilized, by analyzing 16 studies applying state-of-the-art automation in the context of dog behavior analysis. A conceptual model consisting of three steps arose from our analysis: (i) quantification of behavior, (ii) feature extraction, and (iii) hypothesis testing/answering the research question (either using statistical analysis or machine learning). The outcomes of these analyses underscored the necessity of understanding the perceptions and attitudes of animal researchers, particularly in identifying barriers hindering the broader adoption of automation in the field. This realization prompted the initiation of the empirical study. Together, these parts aid in a broader understanding of the current and future use of automation in the field of canine science.

Overall, our analysis reveals a tendency to employ straightforward and basic techniques in both the behavior quantification and feature extraction stages [stages (i) and (ii)] within the proposed conceptual model. This underscores the need for more accessible introductory materials tailored for canine science researchers, providing insights into new methods for both behavior quantification and feature extraction. Additionally, our findings indicate that the testing of hypotheses [stage (iii)] is predominantly addressed through statistical methods. This observation highlights the current reliance on traditional statistical approaches in the canine science research community. However, some recent studies have applied machine learning techniques. By making this distinction explicit, we aspire to spark productive discussions within the canine science, and the more general animal behavior community about the methodological issues related to these complementary data analysis approaches. We also hope this will encourage a greater openness to these alternative approaches among community members.

The above discussion highlights the predominance of basic and straightforward computational methods in current applications, that need to evolve further. However, feedback from the participants in our empirical study points to current challenges in mastering even these computational tools. Many participants expressed concerns over steep learning curves and a general lack of understanding regarding the operation and underlying assumptions of these tools. The relatively small number of participants, as well as using our network to reach them should be acknowledged as a limitation of this study, and future research should include a more representative sample of relevant researchers.

We believe that in today's rapidly evolving research landscape, AI proficiency is becoming increasingly vital for the field of animal behavior. Without this expertise, researchers might struggle to effectively apply AI in their research, potentially missing out on critical findings or misinterpreting AI-generated data. Moreover, a lack of proficiency could lead to a knowledge gap, hindering collaboration with other disciplines and slowing the progress of multidisciplinary research. Therefore, the development of AI education tailored to the needs of this community, as well as fostering multidisciplinary collaboration and dialogue between various communities seem to be the right way forward.

## Data availability statement

The original contributions presented in the study are included in the article/Supplementary material, further inquiries can be directed to the corresponding author.

## Ethics statement

The animal studies were approved by University of Haifa has reviewed the study and waived approval. The studies were conducted in accordance with the local legislation and institutional requirements. Written informed consent was obtained from the owners for the participation of their animals in this study.

## Author contributions

NF: Conceptualization, Data curation, Formal analysis, Investigation, Methodology, Project administration, Software, Supervision, Validation, Visualization, Writing – original draft, Writing – review & editing. AZ: Conceptualization, Formal analysis, Methodology, Validation, Writing – original draft, Writing – review & editing. DV: Conceptualization, Formal analysis, Validation, Writing – original draft, Writing – review & editing. TA: Investigation, Validation, Writing – original draft, Writing – review & editing.
