# Supplementary material for: Automation in canine science: enhancing human capabilities and overcoming adoption barriers
Source: Front Vet Sci. 2024 Jun 14;11:1394620. doi: 10.3389/fvets.2024.1394620 (PMC11212470; doi:10.3389/fvets.2024.1394620)
Supplement: Supplementary file 1 [file Data_Sheet_1.pdf]

Appendix A

A user perceptions study

In this research we aim to study the use of automated analysis tools in the study of animal behavior, focusing on the attitudes of animal researchers towards automated analysis, understanding the pitfalls, best practices, and guidelines and how to navigate this domain moving forward.

Your contribution to this research is highly appreciated and will be immensely valuable in helping us achieve our research goals. We kindly request no more than 15 minutes of your time to complete the survey. Thank you for your participation and support.

Section 1 – Background

- What is your background?
    - Animal behavior.
    - Veterinary Science.
    - Psychology.
    - Cognitive Science.
    - Neuroscience.
    - Other: ____.
- What animal species do you currently investigate?
    - Companion animals (dogs, cats, etc.…).
    - Zoo animals.
    - Wildlife.
    - Laboratory animals.
    - Other: ______.
- Number of years of experience in the field of animal behavior?
    - 0--2 years.
    - 3--5 years.
    - 5--10 years.
    - 10+.

Section 2 - Using an Automated Analysis Tool

- Which automated analysis tool/s have you used? Select all appropriate answers.
    - DeepLabCut.
    - Eztrack.
    - Blyzer.
    - JAABA.
    - BioTracker.
    - Idtracker ai.
    - MARGO.

    - Other: _____.
- Briefly describe your experience with automated tools for data analysis.
- What led you to choose an automated analysis tool for your project/field?

In the following questions, we would like you to reflect on your latest experience working with an automated analysis tool.

- Shortly, describe the analysis task in your project where an automated analysis tool was applied.
- On a scale from 1 to 5, how satisfied are you with the results achieved using the automated analysis tool?
    - Highly satisfied 5 4 3 2 1 Not satisfied at all.
- What challenges did you face while collaborating with data scientists to automatically analyze your data? Please elaborate.
- Was the outcome of the process different from what you expected at the beginning? If so, how?

Section 3 - Obstacles

- How confident are you in understanding the precision of the automated analysis tool and the techniques employed to validate it?
- How much do you trust the outcomes produced by the automated analysis tool? What are the factors that contribute to or detract from your trust?
- What steps can the data scientists take to help you better understand and trust the results?
- What were your difficulties in communication with the data scientists, if at all?
- In hindsight, is there anything you would have done differently in terms of communicating with the data scientists?
- What preprocessing did you need to perform on your data in order to prepare it for automated analysis? How much work was required for this preprocessing, and how significant was its impact on your overall project?
- Did the required preprocessing tasks demand an excessive amount of time and effort in comparison to the achieved outcomes?
- Looking back, is there anything you would have done differently to minimize the need for data preprocessing?
- What benefits has the automated analysis provided for your research?
- Looking back, would you have preferred non-automated analysis methods? If so, why?

Section 4 - Thoughts & Future Work

- [optional] Have you ever attempted to incorporate an automated analysis tool into your research, but were unsuccessful? What were the barriers that prevented successful incorporation?
- In your opinion, what are the barriers to wider adoption of automated analysis tools in your community?
- Do you have any suggestions on how to overcome these barriers?
- How likely are you to use an automated analysis tool in future research?
    - Very likely 5 4 3 2 1 Not likely at all.
- [optional] Did you use other technologies (other than automated data analysis tools) to assist in any previous project? If yes, which?
