# Supplementary material for: Automation in canine science: enhancing human capabilities and overcoming adoption barriers
Source: Front Vet Sci. 2024 Jun 14;11:1394620. doi: 10.3389/fvets.2024.1394620 (PMC11212470; doi:10.3389/fvets.2024.1394620)
Supplement: Supplementary file 2 [file Table_1.pdf]

Table S1: A summary of the reviewed papers, categorized by the discussed topic, research question, behavior quantification method, extracted features, and analysis method.

Study	Ref	Topic	Research Objective	Data Source	Behavior Representation	Extracted Features	Analysis
Bleuer-Elsner et al., 2019	[2]	ADHD-like Behavior	Differences in behavior of dogs diagnosed with ADHD-like behavior after treatment.	Videos	2D Time series of the dog's movement	Movement based features e.g. average speed, covered movement area...	Statistical testing
Aich et al., 2019	[1]	Dog Emotion & Activity Recognition	Differences in behavior across different emotional and activity states.	IMU sensors	3D time series of head and tail	Statistical (mean, minimum, etc.) and peak based (count of peaks, average amplitude, etc.) features	Machine learning
Karl et al., 2020	[9]	Dog-human Relationship	Differences in responses of dogs to human-related stimuli (images of caregiver, stranger, familiar person).	Videos	2D Time series of the dog's movement (relative to two screens)	Residence time in areas of interest.	Statistical testing
Fux et al., 2021	[8]	ADHD-like Behavior	Differences in behavior of dogs diagnosed with ADHD-like behavior vs. control group.	Videos	2D Time series of the dog's movement	Movement based features e.g. average speed, covered movement area.	Machine learning
Byosiere et al., 2022	[4]	Shelter Dogs Welfare	Differences in behavior of shelter dogs before and during COVID.	Videos	2D Time series of the dog's movement	Activity (active/non-active) + step count.	Statistical testing
Ferres et al., 2022	[7]	Dog Emotion Recognition	Differences in behavior in emotional states	Images	24 landmarks on dog body in images.	Pose metrics.	Machine learning
Völter et al., 2022	[13]	Dog-human Relationship	Differences in dog behavior in the unwilling-unable paradigm while transferring food.	Videos	3D time series of the dog's movement	Residence time in areas of interest, visited area of the room, tail angel, head angel.	Statistical testing
Menaker et al., 2022	[10]	Dog Behavioral Testing	Differences in behavior of dogs in a Stranger Test.	Videos	2D Time series of the dog's movement	Movement based features e.g. average speed, covered area, approaching test person.	Machine learning
Boneh Shitrit et al., 2022	[3]	Dog Emotion Recognition	Differences in behavior in emotional states.	Videos	DL deep learning and facial action units.	Learnt from DL	Machine learning
Ren et al., 2022	[11]	Dog-human Relationship	Differences in tail wagging behavior during human interaction.	Videos	3D time series of tail and spine movement	Tail movement based features: angel, amplitude and velocity.	Statistical testing

Study	Ref	Topic	Research Objective	Data Source	Behavior Representation	Extracted Features	Analysis
Wang et al., 2022	[15]	Separation Anxiety	Differences in behavior patterns between normal and separation anxiety symptoms.	IMU sensors	3D time series of head and body	Head posture (head up, head down...) and body posture (walk, stand...) events.	Machine learning
Engelsman et al., 2022	[5]	Ataxia Detection	Differences in walking of dogs diagnosed with ataxia and a control group.	IMU sensors	3D time series of body	Statistical (mean, minimum, etc.) and frequency (mean, peak, etc.) features.	Machine learning
Völter et al., 2023	[14]	Dog-human Relationship	Difference in dog exploratory behavior in presence or separation of caregiver.	Videos	3D time series of the dog's movement (8 key points)	Movement based features: time spent in interest areas, distance traveled, area.	Statistical testing
Farhat et al., 2023	[6]	Dog Behavioral Testing	Differences in behavior of dogs in a Stranger Test.	Videos	2D time series of the dog's movement	Learnt using DL from the time series.	Machine learning
Tsiourti et al., 2023	[12]	Dog Behavioral Testing	Differences in behavior of dogs in a visible and Invisible displacement test of food vs toy.	Videos	2D time series of the dog's movement.	Movement based features: pace, speed, covered movement area.	Statistical testing
Watanangura et al., 2023	[16]	ADHD-like Behavior	Differences in behavior of dogs with ADHD-like behavior before and after treatment.	Videos	2D time series of the dog's movement.	Movement based features: speed, covered movement area, time spent in predefined locations.	Statistical testing

REFERENCES

[1]Satyabrata Aich, Sabyasachi Chakraborty, Jong-Seong Sim, Dong-Jin Jang, and Hee-Cheol Kim. The design of an automated system for the analysis of the activity and emotional patterns of dogs with wearable sensors using machine learning. *Applied Sciences*, 9(22):4938, 2019.

[2]Stephane Bleuer-Elsner, Anna Zamansky, Asaf Fux, Dmitry Kaplun, Sergey Romanov, Aleksandr Sinitca, Sylvia Masson, and Dirk van der Linden. Computational analysis of movement patterns of dogs with adhd-like behavior. *Animals*, 9(12):1140, 2019.

[3]Tali Boneh-Shitrit, Marcelo Feighelstein, Annika Bremhorst, Shir Amir, Tomer Distelfeld, Yaniv Dassa, Sharon Yaroshetsky, Stefanie Riemer, Ilan Shimshoni, Daniel S Mills, et al. Explainable automated recognition of emotional states from canine facial expressions: the case of positive anticipation and frustration. *Scientific reports*, 12(1):22611, 2022.

[4]Sarah-Elizabeth Byosiere, Marcelo Feighelstein, Kristiina Wilson, Jennifer Abrams, Guy Elad, Nareed Farhat, Dirk van der Linden, Dmitrii Kaplun, Aleksandr Sinitca, and Anna Zamansky. Evaluation of shelter dog activity levels before and during covid-19 using automated analysis. *Applied Animal Behaviour Science*, 250:105614, 2022.

[5]Daniel Engelsman, Tamara Sherif, Sebastian Meller, Friederike Twele, Itzik Klein, Anna Zamansky, and Holger A Volk. Measurement of canine ataxic gait patterns using body-worn smartphone sensor data. *Frontiers in veterinary science*, 9:912253, 2022.

[6]Nareed Farhat, Teddy Lazebnik, Joke Monteny, Christel Palmyre Henri Moons, Eline Wydooghe, Dirk van der Linden, and Anna Zamansky. Digitally-enhanced dog behavioral testing. *Scientific Reports*, 13(1):21252, 2023.

[7]Kim Ferres, Timo Schloesser, and Peter A Gloor. Predicting dog emotions based on posture analysis using deeplabcut. *Future Internet*, 14(4):97, 2022.

[8]Asaf Fux, Anna Zamansky, Stephane Bleuer-Elsner, Dirk van der Linden, Aleksandr Sinitca, Sergey Romanov, and Dmitrii Kaplun. Objective video-based assessment of adhd-like canine behavior using machine learning. *Animals*, 11(10):2806, 2021.

[9]Sabrina Karl, Magdalena Boch, Anna Zamansky, Dirk van der Linden, Isabella C Wagner, Christoph J Völter, Claus Lamm, and Ludwig Huber. Exploring the dog–human relationship by combining fmri, eye-tracking and behavioural measures. *Scientific reports*, 10(1):1–15, 2020.

[10]Tom Menaker, Joke Monteny, Lin Op de Beeck, and Anna Zamansky. Clustering for automated exploratory pattern discovery in animal behavioral data. *Frontiers in Veterinary Science*, 9, 2022.

[11]Wei Ren, Pengfei Wei, Shan Yu, and Yong Q Zhang. Left-right asymmetry and attractor-like dynamics of dog's tail wagging during dog-human interactions. *Iscience*, 25(8), 2022.

[12]Christiana Tsiourti, Katie Baynham, Livia Langner, Miriam Ross, Nareed Farhat, Anna Zamansky, and Zsófia Virányi. Comparing the effects of food and toy rewards on canine problem-solving behavior: Insights from the watson task. *Submitted.*, 2023.

[13]Christoph J Völter, Lucrezia Lonardo, Maud GGM Steinmann, Carolina Frizzo Ramos, Karoline Gerwisch, Monique-Theres Schranz, Iris Dobernig, and Ludwig Huber. Unwilling or unable? using 3d tracking to evaluate dogs' reactions to differing human intentions. *bioRxiv*, pages 2022–07, 2022.

[14]Christoph J Völter, Dario Starić, and Ludwig Huber. Using machine learning to track dogs' exploratory behaviour in the presence and absence of their caregiver. *Animal Behaviour*, 197:97–111, 2023.

[15]Huasang Wang, Othmane Atif, Jirong Tian, Jonguk Lee, Daihee Park, and Yongwha Chung. Multi-level hierarchical complex behavior monitoring system for dog psychological separation anxiety symptoms. *Sensors*, 22(4):1556, 2022.

[16]Antja Watanangura, Sebastian Meller, Nareed Farhat, Jan S. Suchodolski, Rachel Pilla, Mohammad R. Khattab, Bruna C. Lopes, Andrea Bathen-Nöthen, Andrea Fischer, Kathrin Busch-Hahn, Cornelia Flieshardt, Martina Gramer, Franziska Richter, Anna Zamansky, and Holger A. Volk. Behavioral comorbidities treatment by fecal microbiota transplantation in canine epilepsy – a pilot study of a novel therapeutic approach. *Submitted.*, 2023.
